# Association between human leukocyte antigen class II (HLA-DRB and -DQB) alleles and outcome of exposure to *Mycobacterium tuberculosis*: a cross-sectional study in Nairobi, Kenya

**DOI:** 10.11604/pamj.2022.41.149.30056

**Published:** 2022-02-21

**Authors:** Susan Odera, Marianne Mureithi, Andrew Aballa, Noel Onyango, Samwel Kazungu, Simon Ogolla, George Kaiyare, Omu Anzala, Julius Oyugi

**Affiliations:** 1 Department of Medical Microbiology and Immunology, University of Nairobi, Nairobi, Kenya,; 2 Kenya Aids Vaccine Initiative (KAVI) Institute of Clinical Research, University of Nairobi, Nairobi, Kenya,; 3 Department of Medical Laboratory Sciences, School of Medicine, Kenyatta University, Nairobi, Kenya,; 4 Department of Clinical Medicine and Therapeutics, School of Medicine, University of Nairobi, Nairobi, Kenya,; 5 Biozeq Kenya Molecular Laboratory, Nairobi, Kenya,; 6 Institute of Tropical and Infectious Diseases, University of Nairobi, Nairobi, Kenya

**Keywords:** Human leucocyte antigen, pulmonary tuberculosis, household contacts, latent tuberculosis, Kenya

## Abstract

**Introduction:**

human leukocyte antigen (HLA) class II alleles play an important role in the early immune response to tuberculosis (TB) by presenting antigenic peptides to CD4+ T cells, hence polymorphisms in those genes can influence the efficiency of the immune response to infection and progression to active disease.

**Methods:**

an analytical cross-sectional study of adult pulmonary tuberculosis (PTB) patients at Mbagathi County Hospital, Nairobi and their HHCs. Sociodemographic data were captured on questionnaires and clinical data extracted from patient files. Intravenous blood samples were drawn for interferon-gamma release assay (IGRA) to determine latent tuberculosis infection (LTBI) among HHCs, and for extraction of DNA used in typing of HLA-DQB1 and HLA-DRB1 alleles by PCR sequence specific primer amplification. Chi-square and Fisher's exact test were used to compare the HLA type II allele frequencies of LTBI negative HHCs, LTBI positive HHCs and active TB patients. Logistic regression was used to adjust for HIV status.

**Results:**

the HLA-DQB1 and HLA-DRB1 alleles were analyzed in 17 PTB and 37 HHCs. Nineteen (19) HHCs were LTBI positive, while 18 were LTBI negative. The frequency of DRB3*1 was 0.17-fold lower [95% CI=0.03-0.83] among PTB patients compared to HHCs before adjustment for HIV status (p=0.048). The frequency of the DRB5*2 allele was significantly higher (p=0.013) among PTB patients (23.5%) compared to HHCS (0.00%). After adjusting for HIV status, the frequency of DRB1*14 was 12-fold higher [95% CI=1.11-138.2] among PTB patients compared to HHCs (p=0.040).

**Conclusion:**

the higher frequencies of HLA-DRB5*2 and HLA-DRB1*14 alleles in PTB patients suggest a likely association with progression to active PTB. The higher frequency of HLA-DRB3*1 allele among LTBI negative HHCs shows its likely protective role against M. tuberculosis infection in this population.

## Introduction

Tuberculosis (TB) continues to be an infectious disease of public health concern, with the highest-burden afflicting sub-Saharan countries. Recent efforts by World Health Organization to reduce and possibly eliminate TB emphasize the importance of treating the pool of individuals with latent TB infection (LTBI) and putting more effort into researching more efficacious population-specific vaccines. Pathogen, host, and environmental factors all play a role in the outcome of exposure to *Mycobacterium tuberculosis*. Although many environmental factors that contribute to the sustained high prevalence of TB are known, it is not clear to what extent host genetic differences may account for the higher levels of infection observed in some countries [1,2]. Infectious diseases exert significant selective genetic pressure, and the genes involved in the immune response are exquisitely diverse [3]. This diversity explains, in part, why some people resist infection more successfully than others [4]. The observations suggest a vital role for host genetic variability in the susceptibility to exogenous pathogens [5]. The frequencies of some host factors, most significantly alleles associated with susceptibility or resistance to TB, may differ among populations according to ethnic or racial backgrounds. Global data shows that TB occurs at different rates among particular races, ethnicities, and families, indicating a genetic predisposition to TB susceptibility [6].

Studies have also shown that certain HLA class II alleles are protective against infection or rapid progression after infection, with a great degree of variation. Several case-control, candidate-gene, family studies, and genome-wide association studies (GWAS) suggest the association of host genetic factors to TB susceptibility or resistance in various ethnic populations. They have reported genetic markers that can predict TB development in human leukocyte antigen (HLA) and non-HLA genes like killer immunoglobulin-like receptor (KIR), toll-like receptors (TLRs), cytokine/chemokines and their receptors, vitamin D receptor (VDR), among others [7]. The HLA class II alleles play an important role in the early immune response to TB by presenting antigenic peptides to CD4 T cells. HLA molecules are among the most polymorphic human gene products known. Polymorphisms affecting antigen processing and presentation, and hence the profile of cytokines secreted, can influence the efficiency of the immune response to infection and can play a significant role in the host response [8]. This diversity would also pose a limitation on the global use of a vaccine candidate if the analysis was based on pathogen strains and allele frequencies of HLA molecules in only specific populations. Therefore, the distribution and frequency of HLA alleles within a certain population would be very useful baseline data for vaccine design studies and in formulation of population-specific intervention strategies, especially in countries classified as having a high TB burden. Here, we analyzed the frequencies of HLA class II alleles of the DRB- and DQB- loci in pulmonary tuberculosis (PTB) patients and compared these frequencies with those of their adult household contacts stratified as LTBI positive and LTBI negative using the Interferon Gamma Assay (IGRA) test. We hypothesized that certain HLA class II alleles might be associated with protection against infection, or upon infection, with failure to progress to active TB.

**Objective:** to evaluate the association between Human Leukocyte Antigen Class II (HLA-DRB and -DQB) alleles frequencies and outcome of exposure to *Mycobacterium tuberculosis*.

## Methods

**Study setting and design:** the study participants consisted of adult PTB patients and their household contacts (HHCs), and all were enrolled at Mbagathi County Hospital in 2016. The hospital is a public health facility under the County Government of Nairobi´s Department of Health Services. It provides a broad range of services, including an outpatient clinic and in-patient wards for PTB patients. All the participants gave informed consent before being enrolled into the study. Thereafter, a study questionnaire was administered after which biological specimens were collected. An analytical cross-sectional design was adopted for this study. Consenting individuals were enrolled using a convenience sampling technique.

### Study population

**Pulmonary tuberculosis (PTB) patients: inclusion and exclusion criteria:** the PTB patients enrolled were adults diagnosed at Mbagathi County Hospital and confirmed to have TB by sputum smear microscopy and GeneXpert test with complementary information from chest X-rays. One hundred and sixty-six (166) PTB patients were enrolled from the outpatient clinic and in-patient TB wards, and 175 HHCs who provided informed consent were also enrolled. Sociodemographic data on age, gender, marital status, level of education, and clinical data such as HIV sero-status was collected using a questionnaire and from patient files. All HHCs were analysed for LTBI. Genotyping of the HLA-DRB and human leukocyte antigen (HLA-DRB) loci was carried out on 20 randomly selected PTB patients´ samples and alleles were successfully identified in 17 patients. Despite repeated analysis of extracted DNA, some of the DNA samples did not yield results in the sequence-specific amplification polymerase chain reaction (SSP-PCR) analysis.

**Household contacts (HHC): inclusion and exclusion criteria:** household contacts were persons living with, shared meals and slept under the same roof with a PTB patient. Adult HHCs were enrolled as they accompanied patients to the out-patient TB clinic or hospital visiting hours in the wards. They showed no signs or symptoms of TB, were apparently in good health, were not pregnant, were not on immunosuppressive drugs, had no history of TB infection and had no prior preventive therapy for LTBI. An IGRA test was used to identify HHCs who had latent TB infection, and based on this, the HHCS were grouped as LTBI positive and LTBI negative. Sociodemographic data was collected using a questionnaire. Confounding factors like race and genetic background in the ethnically heterogeneous urban setting was reduced by recruiting HHCs of the patients, most of whom were relatives of the patient. The presence or absence of HIV in the samples was determined in the laboratory as the IGRA analysis performed. Genotyping of the HLA-DRB and HLA-DRB loci was carried out on 40 randomly selected HHCs´ samples and alleles were successfully identified in 37 HHCs. Despite repeated analysis of extracted DNA, some of the DNA samples did not yield results in the SSP-PCR analysis.

**Blood sample collection:** blood specimen was used to diagnose LTBI from HHCs and for HLA genotyping. For the IGRA test, 5ml of blood was collected aseptically from HHCs through venous puncture into three QuantiFeron tubes (nil, antigen, and mitogen tubes) in the QuantiFeron®-TB Gold in-tube kit (QFT-GIT), (Qiagen, Germany) and transported immediately to the Biozeq- KAVI-ICR laboratory for processing. For HLA typing, 5ml of blood was collected in EDTA blood collection tubes from the PTB patients and the HHCs. The samples were centrifuged at 3000rpm for 10min. Buffy coat were aliquoted into 2ml screw-cap tubes and stored at -20°C till needed for DNA extraction.

**The interferon-gamma release assay (IGRA) test:** the QuantiFeron®-TB Gold in-tube test (QFT GIT) was used to perform the IGRA test according to the manufacturer's (Cellestis Qiagen, Germany) instructions where the HHCs were categorized as LTBI positive and negative based on the Elisa value for interferon gamma (IFN-γ). Diagnosis of LTBI among HHCs was described as reported by Odera *et al*. where the overall prevalence of LTBI was 55.7% [9].

**Extraction of DNA from blood:** deoxyribonucleic acid (DNA) was extracted from the buffy coat of samples using the QIAmp DNA mini kit (qiagen, strasse 1, Hilden Germany) following the manufacturer's instructions. Briefly, 200 μl of the buffy coat was lysed with proteinase K and DNA molecules adsorbed onto silica-gel membranes with a high chaotropic salt concentration. The resulting DNA were washed and eluted in 200 μl of buffer AE and quantified using standard ultraviolet (UV) spectrophotometric analysis. The A260/A280 ratio was between 1.6 - 2.0 via UV spectrophotometry for optimal band visualization.

**HLA-DQB and HLA-DRB genotyping:** the HLA SSP (sequence specific primers) technique was used to identify HLA-DQB1 and -DRB1 alleles from DNA using the PCR method. Genotyping was performed using the -DR and -DQ low resolution kit (Olerup SSP®, AB Franzengatan 5, SE- 112 51 Stockholm, Sweden) following the manufacturer's instructions. The PCR master mix was prepared using PCR water, deoxynucleotide triphosphate (DNTPs), magnesium chloride (MgCl_2_) and Taq-polymerase and aliquoted into 96-well plates with primer mixes. The method allowed simultaneous genotyping of two loci on the same plate (multiplex). The extracted DNA (75ng) was added into each well and PCR amplification done. Gel-electrophoresis was done using a qiaxcel automated machine which uses a special commercial cartridge. The PCR products were displayed on the screen after running for 25 minutes on the Qiaxcel machine. After PCR amplification, the gel picture was uploaded into the Olerup SSP Analysis Software where positive bands were marked in the input data section and analyzed. Genotypes were evaluated using the Olerup SSP HLA allele typing software.

**Statistical analysis:** statistical package for social scientists (SPSS) software (version 25) was used as the statistical analysis tool. The outcomes of exposure to *Mycobacterium tuberculosis* were sterilizing immunity against infection (LTBI negative), clinically latent infection (LTBI positive) and active TB disease. Chi-square test was used to compare HLA alleles frequencies among the infected (PTB patients and LTBI positive HHCs) and the uninfected (LTBI negative HHCs) stratified by gender and HIV status. The Fisher's exact test was used when an expected absolute cell frequency was less than five. Logistic regression was used to control HIV status. Odds ratios (OR) and adjusted odds ratios (AOR) were evaluated as measures of association with a p value <0.05 considered to be statistically significant at 95% confidence interval (CI).

**Ethical considerations:** the Kenyatta National Hospital/the University of Nairobi Ethics and Research Committee (KNH/UoN ERC) reviewed and approved the study, Reference No. KNH-ERC/A/392. Written informed consent was obtained from TB patients and their HHCs before enrolment in the study. Mbagathi District Hospital/Nairobi County granted permission to conduct the study.

## Results

**Demographic and clinical characteristics of the study participants:** we analyzed the DNA extracts of 60 study participants and identified alleles in HLA-DQB and -DRB loci in 54 study participants comprising 17 PTB patients and 37 HHCs. The DNA extracts from the unyielding samples were re-submitted to the typing protocol but it was unsuccessful. The mean age of the HHCs (36.3±11.2 years) was slightly older than that of PTB patients (32.9±9.8 years) although this was not statistically significantly (p=0.26). Most of the participants were female in both the PTB patients and HHCs groups, at 58.8% and 56.8% respectively. More PTB patients had a past history of smoking (5.9%) compared to HHCs (2.7%) while alcohol consumption was higher among the HHCs (16.2%) than the PTB patients (5.9%). The differences in gender, past history of smoking and alcohol consumption were also not statistically significant. We observed that 41.2% of the PTB patients were HIV positive compared to 100% of HHCs who were all HIV negative (p<0.01) (Table 1).

**Table 1:** demographic and clinical characteristics of PTB patients compared to HHCs

Parameters		PTB (17)	HHC (37)	OR (95% CI)	p value
Age in years	Mean±SD	32.9±9.8	36.3±11.2	-	0.26
Gender	Male	7 (41.2)	16 (43.2)	0.99 (0.29- 3.13)	1.00
	Female	10 (58.8)	21 (56.8)	1.09 (0.32-3.41)	1.00
Cigarette smoking		1 (5.9)	1 (2.7)	2.25 (0.11-43.7)	0.53
Alcohol consumption		1 (5.9)	6 (16.2)	0.32 (0.03- 2.44)	0.41
HIV status	Positive	7 (41.2)	0 (0.0)	-	<0.01
	Negative	10 (58.8)	37 (100)	-	<0.01

HIV: human immunodeficiency virus; PTB: pulmonary tuberculosis; HHC: household contacts; OR: odds ratio; CI: confidence interval

**Figure 1:**
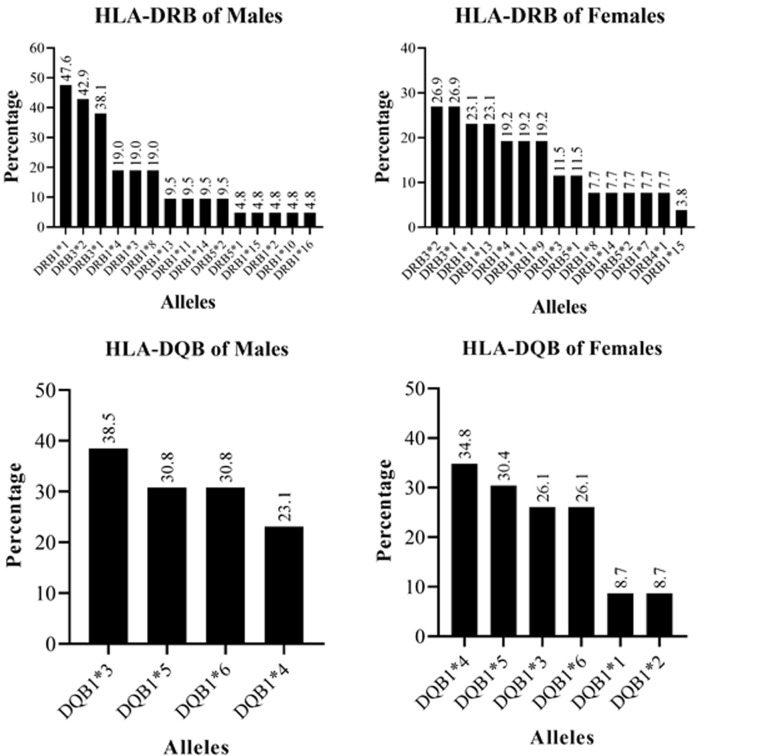
distribution of HLA-DRB and HLA-DQB alleles among the study participants across gender

**Distribution of HLA-DRB and HLA-DQB alleles among the study participants by gender:** from a total of 54 samples analyzed through SSP-PCR genotyping, we determined the HLA-DRB genotypes in 47 samples from which 18 allelic groups were identified while the HLA-DQB genotypes were determined in 36 samples from which 6 allelic groups were identified. We repeated the PCR typing in the unyielding samples for the specific loci but the typing was unsuccessful. HLA-DRB alleles with frequencies >5% were 12 while all identified HLA-DQB alleles had frequencies >5% (Table 2). We compared the frequency of distribution of alleles across gender (Figure 1) and noted that while the frequency most HLA-DRB alleles was higher among males compared to females (12/18), the difference was not statistically significant before adjusting for HIV status (Table 2). The frequency of DQB alleles was comparable among females and males before adjusting for HIV status. After adjustment for HIV status, the frequency of the DRB1*9 allele was significantly higher among females (19.2%) compared to males (0.0), p=0.034 (Table 2).

**Table 2:** distribution of HLA-DRB and HLA-DQB alleles among the study participants by gender

	N	%	Male	Female	p value	Adjusted p value*
DRB alleles	47	100	N=21	N=26		
DRB1*1	16	34.0	10 (47.6)	6 (23.1)	0.122	0.088
DRB3*2	16	34.0	9 (42.9)	7 (26.9)	0.355	0.270
DRB3*1	15	31.9	8 (38.1)	7 (26.9)	0.533	0.428
DRB1*4	9	19.1	4 (19.0)	5 (19.2)	1.000	0.937
DRB1*13	8	17.0	2 (9.5)	6 (23.1)	0.269	0.169
DRB1*3	7	14.9	4 (19.0)	3 (11.5)	0.684	0.476
DRB1*11	7	14.9	2 (9.5)	5 (19.2)	0.436	0.427
DRB1*8	6	12.8	4 (19.0)	2 (7.7)	0.386	0.249
DRB1*9	5	10.6	0 (0.0)	5 (19.2)	0.056	0.034
DRB1*14	4	8.5	2 (9.5)	2 (7.7)	1.000	0.916
DRB5*1	4	8.5	1 (4.8)	3 (11.5)	0.617	0.457
DRB5*2	4	8.5	2 (9.5)	2 (7.7)	1.000	0.096
DRB1*7	2	4.3	0 (0.0)	2 (7.7)	0.495	0.998
DRB1*15	2	4.3	1 (4.8)	1 (3.8)	1.000	0.945
DRB4*1	2	4.3	0 (0.0)	2 (7.7)	0.495	0.998
DRB1*2	1	2.1	1 (4.8)	0 (0.0)	0.447	0.998
DRB1*10	1	2.1	1 (4.8)	0 (0.0)	0.447	0.261
DRB1*16	1	2.1	1 (4.8)	0 (0.0)	0.447	0.998
**DQB alleles**	**36**	**100**	**N=13**	**N=23**		
DQB1*3	11	30.6	5 (38.5)	6 (26.1)	0.474	0.999
DQB1*4	11	30.6	3 (23.1)	8 (34.8)	0.708	0.999
DQB1*5	11	30.6	4 (30.8)	7 (30.4)	1.000	0.416
DQB1*6	10	27.8	4 (30.8)	4 (26.1)	0.679	0.583
DQB1*1	2	5.6	0 (0.0)	2 (8.7)	0.525	0.959
DQB1*2	2	5.6	0 (0.0)	2 (8.7)	0.525	0.959

**Distribution of HLA-DRB and HLA-DQB alleles among pulmonary tuberculosis patients compared to household contacts:** when we compared the distribution of the HLA-DRB and HLA-DQB alleles between the patients and HHCs before adjustment for HIV status, the frequency of the DRB5*2 allele was 23.5% higher among PTB patients compared to HHCS (p=0.013). The frequency of DRB3*1 was 0.17-fold lower (95% CI=0.03-0.83) among PTB patients compared to HHCs before adjustment for HIV status. After adjusting for HIV status, the frequency of DRB1*14 was 12-fold higher (95% CI=1.11-138.2) among PTB patients compared to HHCs (p=0.040). The frequencies of other -DRB and -DQB alleles between PTB patients and HHCs were comparable (Table 3).

**Table 3:** distribution of DRB and DQB alleles among PTB patients and HHCs

	PTB	HHCs	OR (95% CI)	p value	AOR (95% CI) *	p value
DRB alleles	N=17	N=30				
DRB1*1	6 (35.3)	10 (33.3)	1.09 (0.32-3.60)	1.000	1.33 (0.30-5.83)	0.702
DRB1*2	1 (5.9)	0 (0.0)	-	0.361	-	0.998
DRB1*3	1 (5.9)	6 (20.0)	0.25 (0.02-1.93)	0.395	0.00 (0.00-)	0.999
DRB1*4	5 (29.4)	4 (13.3)	2.70 (0.60-9.88)	0.251	2.78 (0.50-15.4)	0.241
DRB1*7	0 (0.0)	2 (6.7)	-	0.528	0.00 (0.00-)	0.999
DRB1*8	3 (17.6)	3 (10.0)	1.92 (0.40-8.99)	0.652	2.25 (0.31-15.9)	0.416
DRB1*9	2 (11.8)	3 (10.0)	1.20 (0.19-6.39)	1.000	0.00 (0.00-)	0.999
DRB1*10	0 (0.0)	1 (3.3)	-	1.000	-	0.999
DRB1*11	2 (11.8)	5 (16.7)	0.66 (0.12-3.97)	1.000	0.00 (0.00-)	0.999
DRB1*13	3 (17.6)	5 (16.7)	1.07 (0.25-5.24)	1.000	2.14 (0.40-11.2)	0.368
DRB1*14	3 (17.6)	1 (3.3)	6.21 (0.82-82.9)	0.127	12.42 (1.11-138.2)	0.040
DRB1*15	0 (0.0)	2 (6.7)	-	0.528	0.00 (0.00-)	0.999
DRB1*16	0 (0.0)	1 (3.3)	-	1.000	-	0.999
DRB3*1	2 (11.8)	13 (43.3)	0.17 (0.03-0.83)	0.048	0.00 (0.00-)	0.999
DRB3*2	3 (17.6)	13 (43.3)	0.28 (0.07-1.08)	0.111	0.14 (0.01-1.296)	0.084
DRB4*1	1 (5.9)	1 (3.3)	1.81 (0.09-35.4)	1.000	3.22 (0.18-56.8)	0.424
DRB5*1	3 (17.6)	1 (3.3)	6.21 (0.82-82.9)	0.127	7.25 (0.58-90.5)	0.124
DRB5*2	4 (23.5)	0 (0.0)	-	0.013	**-**	0.998
**DQB alleles**	**N=14**	**N=22**				
DQB1*1	1 (7.1)	1 (4.5)	1.61 (0.07-31.9)	1.000	0.00 (0.00-)	0.999
DQB1*2	0 (0.0)	2 (9.1)	-	0.511	-	0.999
DQB1*3	4 (28.6)	7 (31.8)	0.85 (0.23-3.26)	1.000	0.71 (0.11-4.47)	0.719
DQB1*4	6 (42.9)	5 (22.7)	2.55 (0.64-11.6)	0.273	2.04 (0.35-11.67)	0.423
DQB1*5	5 (35.7)	6 (27.3)	1.48 (0.34-5.83)	0.715	1.60 (0.28-8.85)	0.590
DQB1*6	3 (21.4)	7 (31.8)	0.58 (0.14-2.45)	0.706	0.71 (0.11-4.47)	0.719

PT: pulmonary tuberculosis; HHCs: household contacts; HLA: human leukocyte antigen; OR: odds ratio; CI: confidence interval; AOR: adjusted odds ratio; *adjusted for HIV status of participants

**Figure 2:**
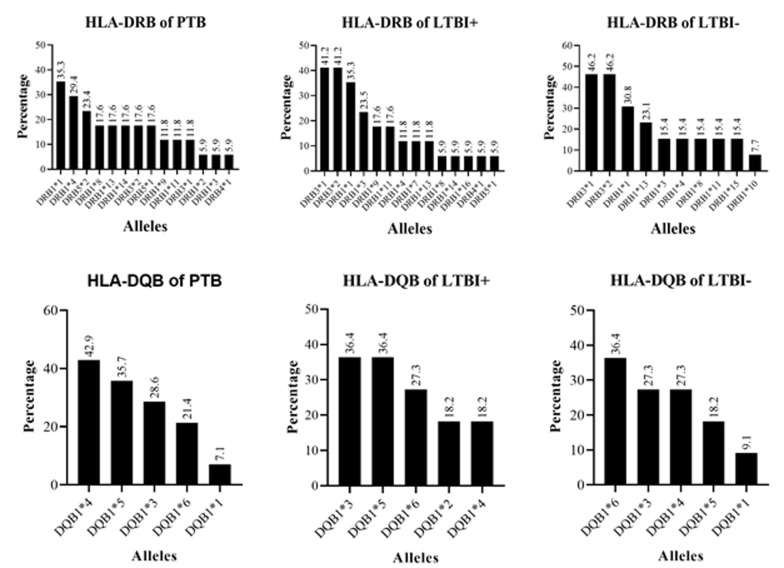
illustration of the distribution of HLA-DRB and HLA-DBQ alleles between PTB patients, LTBI positive HHCs and LTBI negative HHCs

**Distribution of HLA-DRB and HLA-DQB alleles among study participants stratified as pulmonary tuberculosis patients, latent TB negative household contacts, and latent TB positive household contacts:** allele distribution between PTB, LTBI-ve, and LTBI +ve HHCs was compared (Figure 2). The results of the IGRA test revealed 19 HHCs were infected (latent TB positive) while 18 were uninfected (latent TB negative). The frequency of the HLA-DRB3*1 allele was 34.4% significantly higher among LTBI -ve HHCs than PTB patients (p=0.049) before adjustment for the HIV status of patients. The frequency of other DRB alleles and all the DQB alleles evaluated was comparable between PTB and LTB+, PTB and LTBI -ve and LTBI +ve and LTBI -ve. After adjusting for HIV status, the frequency of -DRB and -DQB alleles was comparable between PTB and LTBI+, PTB and LTBI -ve and LTBI +ve and LBVI -ve (Table 4).

**Table 4:** distribution of HLA-DRB and HLA-DBQ alleles between PTB patients, LTBI positive HHCs and LTBI negative HHCs

	Group			p value			Adjusted p value*		
	PTB	LTB+	LTB-	PTB/ LTB+	PTB/ LTB-	LTB+ /LTB-	PTB/ LTB+	PTB/ LTB-	LTB+ /LTB-
DRB alleles	N=17	N=17	N=13						
DRB1*1	6 (35.3)	6 (35.3)	4 (30.8)	1.000	1.000	1.000	0.807	0.646	0.795
DRB1*2	1 (5.9)	0 (0.0)	0 (0.0)	1.000	1.000	-	0.998	0.999	-
DRB1*3	1 (5.9)	4 (23.5)	2 (15.4)	0.335	0.564	0.672	0.999	0.999	0.583
DRB1*4	5 (29.4)	2 (11.8)	2 (15.4)	0.398	0.426	1.000	0.253	0.407	0.773
DRB1*7	0 (0.0)	2 (11.8)	0 (0.0)	0.484	-	0.492	0.050	-	0.999
DRB1*8	3 (17.6)	1 (5.9)	2 (15.4)	0.601	1.000	0.564	0.286	0.773	0.406
DRB1*9	2 (11.8)	3 (17.6)	0 (0.0)	1.000	0.492	0.237	0.999	1.000	0.999
DRB1*10	0 (0.0)	0 (0.0)	1 (7.7)	-	0.433	0.433	-	0.999	0.998
DRB1*11	2 (11.8)	3 (17.6)	2 (15.4)	1.000	1.000	1.000	0.999	0.999	0.869
DRB1*13	3 (17.6)	2 (11.8)	3 (23.1)	1.000	1.000	0.627	0.253	0.708	0.417
DRB1*14	3 (17.6)	1 (5.9)	0 (0.00	0.601	0.237	1.000	0.121	0.999	0.999
DRB1*15	0 (0.0)	0 (0.0)	2 (15.4)	-	0.179	0.179	-	0.999	0.998
DRB1*16	0 (0.0)	1 (5.9)	0 (0.0)	1.000	-	1.000	0.999	-	0.999
DRB3*1	2 (11.8)	7 (41.2)	6 (46.2)	0.117	0.049	1.000	0.999	0.999	0.785
DRB3*2	3 (17.6)	7 (41.2)	6 (46.2)	0.258	0.123	1.000	0.114	0.087	0.785
DRB4*1	1 (5.9)	1 (5.9)	0 (0.0)	1.000	1.000	1.000	0.696	0.999	0.999
DRB5*1	3 (17.6)	1 (5.9)	0 (0.0)	0.601	0.237	1.000	0.286	0.999	0.999
DRB5*2	4 (23.4)	0 (0.0)	0 (0.0)	0.102	0.112	-	0.998	0.999	-
**DQB alleles**	**N=14**	**N=11**	**N=11**						
DQB1*1	1 (7.1)	0 (0.0)	1 (9.1)	1.000	1.000	1.000	1.000	0.999	0.999
DQB1*2	0 (0.0)	2 (18.2)	0 (0.0)	0.183	-	0.476	0.999	-	0.999
DQB1*3	4 (28.6)	4 (36.4)	3 (27.3)	1.000	1.000	1.000	0.601	0.912	0.648
DQB1*4	6 (42.9)	2 (18.2)	3 (27.3)	0.233	0.676	1.000	0.353	0.657	0.613
DQB1*5	5 (35.7)	4 (36.4)	2 (18.2)	1.000	0.406	0.635	0.960	0.353	0.346
DQB1*6	3 (21.4)	3 (27.3)	4 (36.4)	1.000	0.656	1.000	0.912	0.601	0.648

PTB: pulmonary tuberculosis; LTB+: latent TB positive; LTB-: latent TB negative; *adjusted for HIV status

## Discussion

The overall goal of this study was to evaluate the association between HLA-DRB and HLA-DQB alleles frequencies and susceptibility to *Mycobacterium tuberculosis* infection in a Kenyan population. The study participants' mean age and gender distribution was comparable across the patients and HHCs. The mean age of the PTB patients (32.9 years) mirrored the 2019 WHO report of TB in Kenya which documented that the majority of TB infected individuals are in the 25-34 age group (WHO 2019 TB global data report Kenya). Analysis of HLA-DRB locus revealed -DRB1*1 (34.0%) and -DRB3*2 (34.0%) alleles were the highest in the population, followed by -DRB3*1 (31.9%) and -DRB1*4 (19.1%) while the least frequent alleles were -DRB1*2, DRB1*10 and DRB1*16 (all occurring at 2.1%). At the DQB locus, DQB1*3, DQB1*4 and DQB1*6 were the most frequent, being identified among 30.6% of the study population DQB1*1and DQB1*2 were the least frequent (both occurring at 5.6%). A 2014 study report on the diversity of HLA class I and II alleles in an East African population also identified HLA-DRB3*2 and HLA-DQB1*3 among the most common alleles in the -DRB and -DQB loci [10]. Similar to our study, only one HLA-DRB4 allele (DRB4*1) was identified in both study populations. However, observed differences in frequency of other alleles in the two studies could be due to variations in the ethnic background of the populations in the different geographical locations and the gender bias of female participants only in the East African study. A comparison of the frequency distribution of the alleles across gender revealed an even distribution of HLA-DQB alleles. Analysis of the distribution of the HLA-DRB alleles revealed a higher frequency in males compared to females except the HLA-DRB1*9 alleles which was significantly higher (p=0.034) among females after controlling for the HIV sero-status. Few studies have suggested that gender significantly influences the distribution of HLA-DR and -DQ alleles [11], and more research on our study finding should be explored in a larger study.

Comparative analyses were done and frequencies and odds ratios interpreted as the measure of association between HLA allele frequency and susceptibility to tuberculosis infection and/or progression to active TB. This study did not find significant differences in the frequencies of HLA-DQB alleles in the PTB patients, latent tuberculosis-infected, and latent tuberculosis negative HHCs. Therefore, none of the identified HLA- DQB alleles were associated with infection susceptibility or progression to PTB in this population. This finding was in agreement with the study of Duarte *et al*. in 2011 who studied a Portuguese population and found no association between the phenotypic distribution of HLA-DQB in the healthy exposed group (healthy exposed positive and healthy exposed negative) and patients who had active TB [12]. Other studies by Cao *et al*. in the USA and Lombard *et al*. in South Africa also reported no association, complementing our findings [13,14]. However, a study in Uganda reported a negative correlation between TB susceptibility and the DQB1*3 allele, which contradicted our findings [15]. Additionally, other studies in India [16], Thailand [17], and Iran [18] reported a positive correlation between TB susceptibility and expression of DQB alleles. From the available data, the divergent associations might be due to differences in sample size, study designs, and the genetic heterogeneity of the *Mycobacterium tuberculosis* complex, which were not controlled during our analyses. We also had a small sample size that might have underestimated or overestimated the link between the HLA-DQB1 alleles and TB susceptibility or resistance in this population.

The HLA-DRB3*1 allele occurred at a statistically significant higher frequency (p=0.049) among latent TB negative HHCs (46.2%) compared to PTB patients (11.8%). The negative association with susceptibility suggests that this allele could be protective against infection and progression to active TB. Our findings contradicted a 2019 study in Mali, West Africa which reported the HLA-DRB3*01: 01 allele among others had significant association with *Mycobacterium tuberculosis* infection compared to healthy controls [19]. Unlike our study which did not control for the strains in the *Mycobacterium tuberculosis* complex, the Mali study analyzed whether the association of HLA with TB susceptibility was strain dependent with epidemiological evidence that *M. africanum* was more common in West Africa compared to other regions in the continent. Furthermore, the variation in HLA-DRB allele distribution across populations in Africa's Western and Eastern regions could also contribute to the different findings. Because of the paucity of published literature on the association of the HLA-DRB3*1 allele with TB disease, we could not make further comparisons with our findings. Notably, the DRB3*1 allele was one of the most common HLA-DRB alleles occurring in 31.9% of our study population. Pathogens often adapt to the most frequent HLA alleles, while rarer ones have a selective advantage because of host-pathogen interactions [20], which could explain why the most frequent alleles in the study population were not linked with TB susceptibility/progression. Furthermore, the HLA-DRB5*2 allele occurred at a statistically significant (p=0.013) higher frequency among PTB patients compared to HHCs. Although its frequency was low in our study population (8.5%), the higher occurrence in PTB patients (23.5%) compared to HHCs (0.0%), hints at a positive association with T.B. progression and/or susceptibility to infection. Our finding was in agreement with a 2017 study of susceptibility loci associated with tuberculosis in Han Chinese which reported that candidate genes that were significantly associated with TB included among others genes in the HLA-DRB5 [21]. The HLA-DRB5*2 allele may have the “rare allele advantage” in this population which argues that pathogens adapt to HLA alleles that are more prevalent in the population, thus such alleles would not have a significant role to play in the immune-pathogenicity of those pathogens [22].

All HHCs in this study population were HIV negative, while 41.2% of the PTB patients were HIV positive. HIV infection is a known factor that increases the risk of progression to active TB. Therefore, during statistical analysis on the distribution of the HLA alleles, the HIV sero-status of the study participants was controlled for. After controlling for HIV status in our study population, the HLA-DRB1*14 alleles were significantly higher (p=0.040) among the PTB patients compared to HHCs, suggesting its likely contribution to development of active TB. This finding was in agreement with a study by Duarte *et al*. in 2011 in a Portuguese population where the frequency of the HLA-DRB1*14 allele was significantly higher in TB patients compared to healthy exposed controls [12]. Similar reports from India [23] and Russia [24] also suggested that this could be a susceptibility allele for evolution from infection to active TB. The role of the HLA-DRB1*14 allele in TB immunopathogenesis is also highlighted in a study on serology-based antibodies for TB diagnosis where researchers documented a significant increase in the frequency of HLA-DRB1*14 among subjects with high antibody response levels compared to those with low levels [25]. Our findings suggest the HLA-DRB5*2 and HLA-DRB1*14 alleles may be risk factors for progression to active TB in our study population. The HLA-DRB5*2 had a positive association with progression to active TB before controlling for HIV sero-status while the HLA-DRB1*14 alleles were a significant factor in development of active TB after controlling for HIV sero-status. The chances of an individual with recently acquired T.B. infection progressing to active disease are higher with HIV coinfection. Many studies postulate the likely role of HLA polymorphisms in the differential development of TB in HIV infected individual [26-28]. Furthermore, a study conducted in China highlighted the important role that the HLA-DRB1*14 plays in accelerated disease progression in HIV infection [29]. Closely related to this, our study findings suggest that in our study population, the HLA-DRB1*14 allele plays a synergistic role in disease progression in an individual exposed to both HIV and *Mycobacterium tuberculosis*.

## Conclusion

This study provides an important insight into the nature of HLA class II alleles and *Mycobacterium tuberculosis* infection and active TB in this population. The findings suggest the HLA-DRB5*2 and HLA-DRB1*14 alleles may be risk factors for developing active TB. Although the varied contribution of each allele to risk of latent or active TB before and after controlling for HIV is open to further research, both alleles would make natural candidates for further research on genetic susceptibility to TB. The study also highlights the potential protective role that the HLA-DRB3*1 allele may have against *Mycobacterium tuberculosis* infection. However, the evidence from this study is limited by the small sample size. We recommend further studies to investigate the roles of these alleles in TB immunopathogenesis as predictive biomarkers of infection outcome and vaccine candidate development in this population.

### What is known about this topic

- *The human leucocyte antigen (HLA) class II alleles play an important role in the early immune response to tuberculosis (T.B.) by presenting antigenic peptides to CD4+ T cells, hence polymorphisms in those genes can influence the efficiency of the immune response to infection and progression to active disease;*
- *The nature and localization of HLA polymorphism observed in populations is likely to be functionally significant in terms of disease susceptibility because different populations exhibit frequency distribution of alleles and extended haplotypes particular to that group*.

### What this study adds

- *This study contributes to the pool of knowledge on the distribution of the HLA-DRB and HLA-DQB alleles in this population, by highlighting the most frequent and least frequent alleles;*
- *This study also provides important insight into the HLA class II alleles of the -DRB locus that would make natural candidates for further research on genetic susceptibility to T.B. in this population*.
